# Supplementary material for: EPO-R76E Enhances Retinal Pigment Epithelium Viability Under Mitochondrial Oxidative Stress Induced by Paraquat
Source: Cells. 2025 Nov 14;14(22):1794. doi: 10.3390/cells14221794 (PMC12651157; doi:10.3390/cells14221794)
Supplement: Supplementary file 1 [file cells-14-01794-s001.zip › cells-3891767-supplementary.pdf]

Supplementary figures

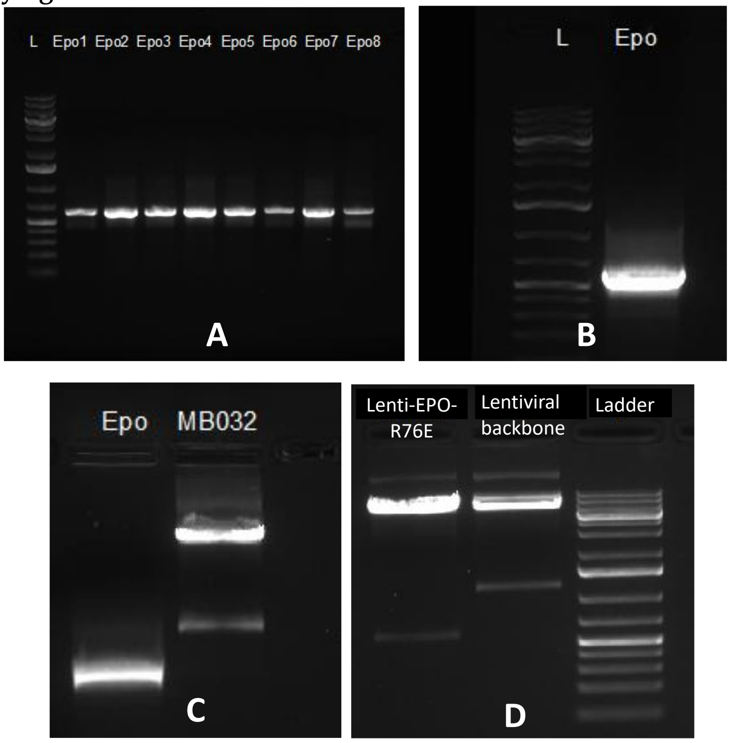

Figure S1. Preparation and Characterization of Lenti-EPO-R76E Plasmid. (A) Gradient PCR amplification of EPO-R76E using EcoRI and NotI primers. A distinct band at ~600 bp was observed across varying annealing temperatures, indicating successful amplification. (B) Agarose gel showing pooled PCR product prior to gel extraction. The ~600 bp band was excised and purified for downstream cloning. (C) Agarose gel electrophoresis of EcoRI and NotI restriction-digested EPO-R76E insert (Epo) and lentiviral vector backbone (MB032). Distinct bands corresponding to the ~600 bp insert and ~7000 kb vector confirm successful digestion and preparation for ligation. (D) Agarose gel electrophoresis of the plasmid digestion with restriction enzymes EcoRI and NotI, yielding expected fragment sizes (7072 bp and 582 bp), confirming correct insertion of the EPO gene into lentiviral vector.

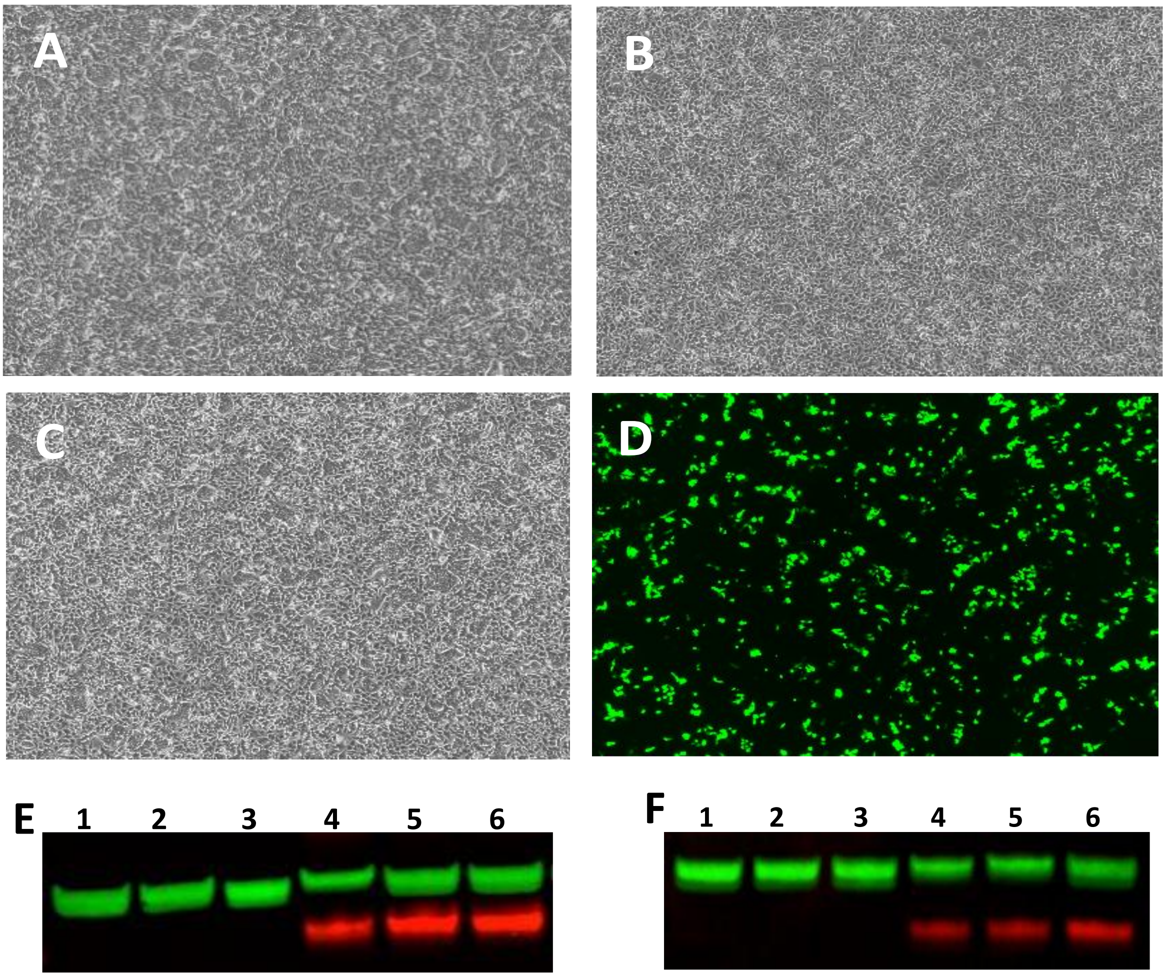

Figure S2. Validation of Lenti-EPO-R76E Transfection in HeLa and ARPE-19 Cells. (A) Brightfield image of non-transfected HeLa control cells (B) Brightfield image of HeLa cells transfected with Lenti-EPO-R76E plasmid using PEI (C) Brightfield image of HeLa cells transfected with GFP plasmid, used as a transfection efficiency control, (D) Fluorescence image of GFP-transfected HeLa cells taken 24 hours post-transfection, demonstrating strong green fluorescence signal, confirming successful transfection. (E) Western Blot Analysis of EPO-R76E Expression in Transfected HeLa Cells. Western blot was performed on protein lysates collected 24 hours post-transfection using anti-2A antibody. A specific band (red bands) corresponding to EPO-R76E was detected at ~26 kDa in HeLa cells transfected with Lenti-EPO-R76E (Lane 4, 5, 6). No EPO band was observed in either the GFP-expressing plasmid transfected (Lane 2, 3) or non-transfected control (Lane 1) cells. β-actin (~42 kDa) was used as a loading control (green bands). (F) Validation of stable EPO-R76E expression in ARPE-19 cells by Western blot. Protein lysates from ARPE-19 (Lane 1, 2, 3) and stable ARPE-19-EPO-R76E (Lane 4, 5, 6) cells were analyzed by Western blot using a polyclonal anti-EPO antibody (red) and anti-β-actin antibody (green) as a loading control. A specific EPO band was detected at ~26 kDa only in the EPO-R76E-expressing cells. β-actin (~42 kDa) was consistently detected in both samples. Fluorescent detection was performed using LI-COR dual-channel imaging.
